# Structure-based dynamic analysis of the glycine cleavage system suggests key residues for control of a key reaction step

**DOI:** 10.1038/s42003-020-01401-6

**Published:** 2020-12-11

**Authors:** Han Zhang, Yuchen Li, Jinglei Nie, Jie Ren, An-Ping Zeng

**Affiliations:** 1 Beijing Advanced Innovation Center for Soft Matter Science and Engineering, Beijing University of Chemical Technology, North Third Ring Road 15, Chaoyang District, 100029 Beijing, China; 2 State Key Laboratory for Biology of Plant Diseases and Insect Pests/Key Laboratory of Control of Biological Hazard Factors (Plant Origin) for Agri-product Quality and Safety, Ministry of Agriculture, Institute of Plant Protection, Chinese Academy of Agricultural Sciences, 100081 Beijing, China; 3 Hamburg University of Technology, Institute of Bioprocess and Biosystems Engineering, Denickestrasse 15, D-21073 Hamburg, Germany

**Keywords:** Enzyme mechanisms, Molecular modelling

## Abstract

Molecular shuttles play decisive roles in many multi-enzyme systems such as the glycine cleavage system (GCS) for one-carbon (C1) metabolism. In GCS, a lipoate swinging arm containing an aminomethyl moiety is attached to protein H and serves as a molecular shuttle among different proteins. Protection of the aminomethyl moiety in a cavity of protein H and its release induced by protein T are key processes but barely understood. Here, we present a detailed structure-based dynamic analysis of the induced release of the lipoate arm of protein H. Based on molecular dynamics simulations of interactions between proteins H and T, four major steps of the release process showing significantly different energy barriers and time scales can be distinguished. Mutations of a key residue, Ser-67 in protein H, led to a bidirectional tuning of the release process. This work opens ways to target C1 metabolism in biomedicine and the utilization of formate and CO_2_ for biosynthesis.

## Abstract (web summary)

Han Zhang et al. report molecular dynamics simulations and mutational analysis of a key process of the *E. coli* glycine cleavage system, an important enzyme complex in C1 metabolism. They identify a key amino acid residue controlling the release of the swinging aminomethyl lipoate arm and increase the overall reaction rate of glycine cleavage by more than twice, providing a strategy for manipulating this reaction system for use in synthetic biology.

## Introduction

The swinging arms of molecular shuttles play decisive roles in many essential biological reaction systems, such as the glycine cleavage or synthesis system, pyruvate and alpha-oxoglutarate dehydrogenase complexes, and the branched-chain amino acid dehydrogenase complex^1–3^. Swinging arms shuttle functional groups or molecular moieties among different parts of these enzyme complexes^1^. When bound with a molecular moiety, a swinging arm is normally protected in the cavity of a shuttle protein during a certain period of the catalytic cycle for stability and efficiency. Despite the fundamental importance of this process, quantitative and dynamic studies of the protection and release processes of molecular swinging arms have seldom been reported and targeted engineering or redesign of molecular shuttles remains challenging. In this work, guided by molecular dynamics simulations, we present for the first time detailed information about the breaking and tuning of the protection of the lipoate swinging arm of shuttle protein H in the glycine cleavage system (GCS).

**Fig. 1:**
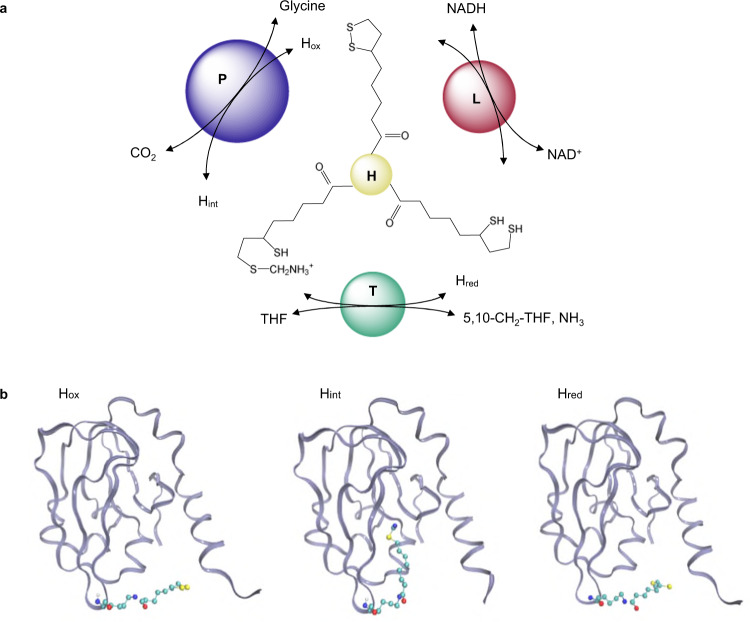
Glycine cleavage system (GCS) and the three forms of protein H. **a** GCS showing the shuttle protein H and the molecules and reactions catalyzed by proteins P, T, and L, respectively. THF tetrahydrofolic acid, H_ox_ an oxidized form of protein H, H_int_ an intermediate form of protein H, H_red_ reduced form of protein H. **b** Structures of the H_ox_, H_int_, and H_red_ from *Pisum sativum*^22^.

GCS cleaves glycine into CO_2_ and NH_3_, yielding reducing power (NADH) and a one-carbon (C1) unit bound to tetrahydrofolate (THF) (Fig. 1a). It plays central roles in C1 metabolism and the biosynthesis of purines and nucleotides^4^. For instance, the deficient activity of GCS leads to nonketotic hyperglycinemia for which no drugs are currently available^5^. Recent studies suggested that controlled and reproducible changes in C1 metabolism are desired in anti-aging and anti-obesity therapies, a variety of cancers^6,7^, and managing infection by certain pathogenic bacteria^8,9^. Therefore, fundamental studies on the reaction mechanism of GCS are essential for developing small-molecule drugs for the treatment of such diseases. Recently, with C1 synthetic biology being regarded as a promising new direction for biological uses of CO_2_ and formic acid^10–12^, GCS has also become a focus for biotechnology research^13,14^. The reductive glycine pathway (rGP) is considered to be the most promising pathway for the biosynthetic use of formate, which can be effectively derived from CO_2_^15^.

GCS was discovered and intensively studied in the 1960–1990’s^16–19^. It is composed of four proteins: H, P, T, and L. The four proteins are separable, but catalyze a coordinated set of reactions (Fig. 1a). A lipoate arm is attached to protein H and serves as a molecular shuttle among the other three proteins. It links the activities of proteins P, T, and L in a cyclic manner, during which it goes through three forms: intermediate (H_int_), reduced (H_red_), and oxidized (H_ox_)^20,21^, as illustrated in Fig. 1b. The lipoate arm in its H_ox_ and H_red_ forms is either located on the surface of protein H or is partially movable in an aqueous solution. However, the lipoate arm with an aminomethyl moiety (H_int_) attached is normally bound and thus protected in a cavity of protein H before it is reduced by protein T^2^.

Experimental data indicated that H_int_ hardly reacts with THF in the absence of protein T^22^. The addition of protein T can rapidly increase the rate of reaction between H_int_ and THF. It is interesting to note that formaldehyde could be produced from the aminomethyl moiety on protein H in the presence of protein T but in the absence of THF^22^. Literature results^22,23^ suggest that the aminomethyl moiety undergoes a facile nucleophilic attack by OH^−^ in an aqueous solution. Since formaldehyde is very toxic for (micro)organisms because it crosslinks proteins and DNA, evolution has developed a fascinating self-protection mechanism for the aminomethyl moiety: it is “hidden” in the hydrophobic cavity of H_int_ until the interaction with protein T occurs and the reaction with THF can take place (Fig. 1b). Previous studies further suggested that the N-terminal region of protein T is essential for inducing conformational changes in protein H when protein T interacts with protein H^19,24^, but how protein T induces changes in the conformation of protein H and how the aminomethyl lipoate arm is released have not yet been clarified. This fundamental question is hard to elaborate via static structural analysis.

In this work, we first examined the induction of conformational changes in protein H by protein T using molecular dynamics (MD) simulations and identified key steps and amino acid residues involved in the release of the aminomethyl lipoate arm. Umbrella sampling was used to analyze the conformational changes of proteins H and T. Mutagenesis experiments were carried out to confirm a key amino acid residue identified for the release of the aminomethyl lipoate arm. The results reveal important molecular details about a key reaction step of GCS and pave the way for a purposeful manipulation of a fundamentally important enzyme complex in C1 metabolism.

## Results

### MD simulations and the protected state of ecH_int_

**Fig. 2:**
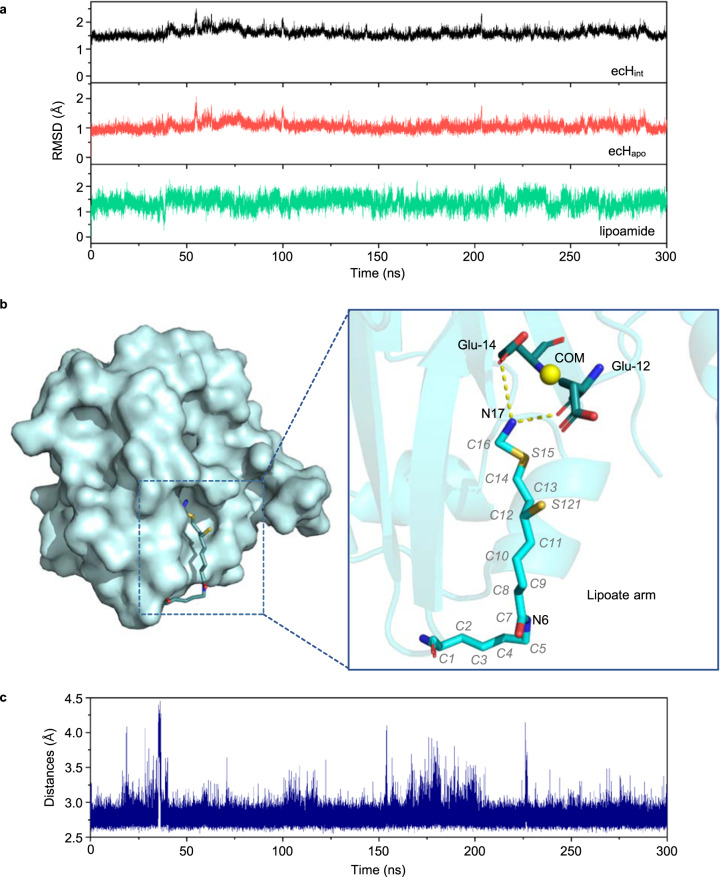
Molecular dynamic simulations of protein H. **a** Root-mean-square deviations (RMSD) calculated for ecH_int_, ecH_apo_, and aminomethyl lipoate arm. **b** Hydrogen-bond interactions among the lipoate arm and the surrounding amino acid residues. N17 in the aminomethyl lipoate arm forms hydrogen bonds with Glu-12 and Glu-14 of protein H and renders thereby the aminomethyl lipoate arm in the cavity of protein H in a stable state. **c** Distance between N17 located in the aminomethyl lipoate arm and the COM of Glu-12 and Glu-14.

After setting up a structural model for *Escherichia coli* H_int_ (ecH_int_) (see “Methods”), we carried out MD simulations of ecH_int_ for 300 ns to check its stability. The root-mean-square deviations (RMSD) of backbone heavy atoms of ecH_int_, ecH_apo,_ and aminomethyl lipoate arm in the system are shown in Fig. 2a. The average RMSD of ecH_int_ turned out to be 1.59 ± 0.13 Å, and the average RMSD values for ecH_apo_ and the aminomethyl lipoate arm were calculated to be 1.05 ± 0.13 Å and 1.37 ± 0.23 Å, respectively. These results indicate that the structure of ecH_int_ is stable, and the aminomethyl lipoate arm is in a stable state protected by the cavity of protein H through hydrogen-bond interactions (Fig. 2b). Moreover, we calculated the distance between N17 located in the aminomethyl lipoate arm and the center of mass (COM) of Glu-12 and Glu-14 as a determinant for the interaction between the aminomethyl lipoate arm and the cavity. The distance between N17 and COM is concentrated between 2.5 Å and 3.0 Å (Fig. 2c). This means that the aminomethyl lipoate arm is locked into a highly stable conformation by protein H, thereby preventing the aminomethyl moiety from nucleophilic attack by OH^−^ in solution, until protein T binds to protein H and induces the release of the aminomethyl lipoate arm.

### Induced release of the aminomethyl lipoate arm via interactions with protein T

**Fig. 3:**
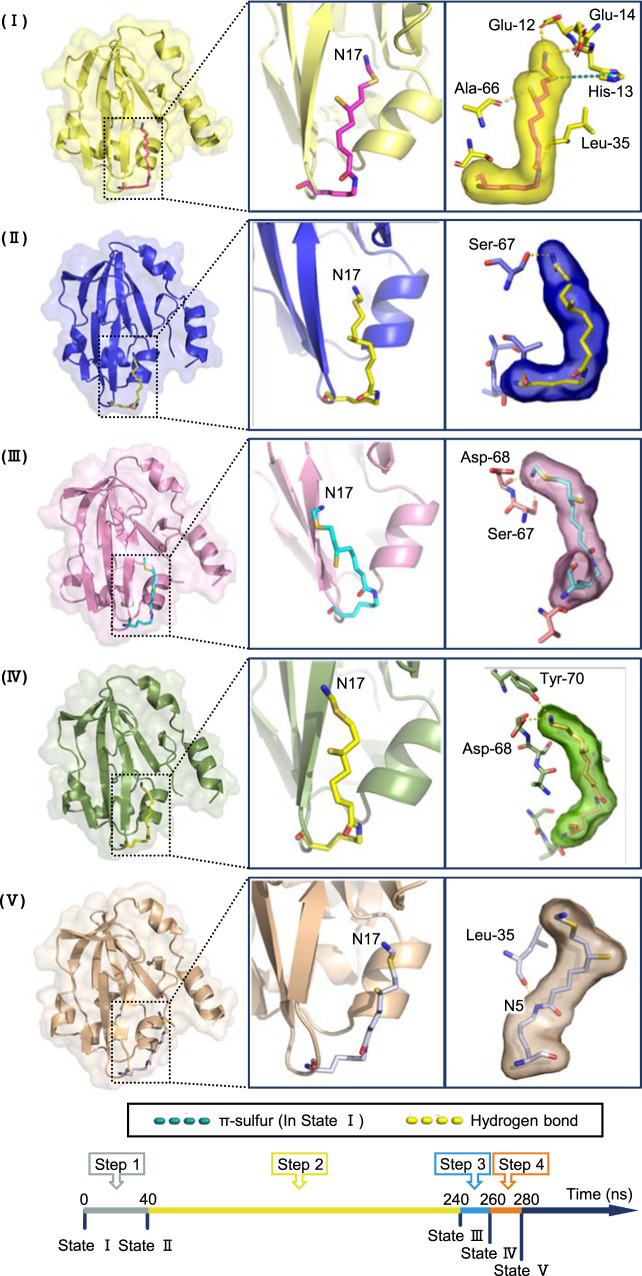
Five distinguished states of the release process of the aminomethyl lipoate arm from the cavity of protein H which divide the whole process into four major steps. State V defines the time point at which the aminomethyl lipoate arm completely leaves the cavity of protein H. Time spent (in nanoseconds) in each step is summarized in the bottom panel. In panel I (State I), hydrogen bonds and π-sulfur interactions at the beginning are indicated.

We carried out MD simulations to study the interactions of ecH_int_ and ecT which lead to a release of the aminomethyl moiety from its protected state. Overall, protein T induces conformational changes in ecH_int_, which in turn causes the aminomethyl lipoate arm to be released from the cavity in protein H within 280 ns (Fig. 3). The dynamic process is visualized in the Supplementary Materials (Supplementary Fig. 1). We paid special attention to interactions between the aminomethyl moiety and residues in the hydrophobic cavity of protein H during the release process. For this purpose, we analyzed the contact of every heavy atom of the lipoic arm with protein residues in the hydrophobic cavity. In this regard, hydrogen bonds formed between nitrogen atoms (N6 and N17) of the aminomethyl lipoate arm and residues located in the cavity are found to play dominant roles. Since these nitrogen atoms are charged, they form so-called “charged hydrogen bonds” with hydrogen atoms of certain residues in the cavity. The energy of a charged hydrogen bond can reach as high as 137.7 kJ/mol compared to merely 17–40 kJ/mol for an ordinary hydrogen bond^25^ and usually less than 10 kJ/mol for a single van der Waals contact. The formation of charged hydrogen bonds between the aminomethyl lipoate arm and residues in the cavity was identified by a detailed examination of MD simulation trajectories, in which each time frame of the trajectory was inspected for the formation and breaking of hydrogen bonds (Supplementary Fig. 2). Three residues Ser-67, Asp-69, and Tyr-70 were found to play dominant roles in this respect. Detailed information about the charged hydrogen bonds identified is summarized in Supplementary Table 1.

According to our temporal analysis of the formation of charged hydrogen bonds between the different acceptors and donors, five distinct conformational states of the release process can be ascertained which divides the process into four major steps (Fig. 3). State I represents the beginning of the first step, in which the aminomethyl lipoate arm is locked into the cavity of protein H mainly by forming charged hydrogen bonds with the polar residues there, e.g., Glu-12 and Glu-14. Besides, the ordinary hydrogen bond between S121 and Ala-66, and the π–sulfur interaction between S15 and His-13 also stabilize the embedded structure. In our simulations of the interactions between ecHint and ecT-protein, this pseudostable state of ecH_int_ persists for about 40 ns. After that, due to the presence of protein T, the N17 atom on the aminomethyl moiety of the lipoate arm moves away from residues Glu-12 and Glu-14 and forms a hydrogen bond with residue Ser-67 in the β7 sheet of protein H (State II). This interaction strongly hinders the release of the aminomethyl lipoate arm. As the induction of protein T proceeds, the interactions between Ser-67 and the aminomethyl moiety become ever weaker and residue Asp-68 takes over as the main interaction partner of the aminomethyl moiety (State III). The second step (from State II to State III) lasts for 200 ns, taking up more than two-thirds of the total time of the release process. Subsequently, the aminomethyl lipoate arm leaves via the edge of the cavity, and State IV is reached, in which Tyr-70 forms the main hydrogen-bond interaction with the aminomethyl moiety. Although the third step (from State III to State IV) only takes ~20 ns, it makes a significant contribution to the release process. In this step, most atoms of the aminomethyl lipoate arm have already left the cavity of protein H, representing the most significant conformational change. Accompanying the leaving of the aminomethyl lipoate arm, the interactions that held the aminomethyl lipoate arm in the cavity starts to fade away, and hydrogen bonding between the N6 atom in the aminomethyl lipoate arm and residue Leu-35 of protein H becomes the next hallmark (State V). This step lasts also about 20 ns and plays a supporting role in the process of releasing the aminomethyl lipoate arm from the cavity. As the induction of protein T continues, hydrogen-bond interactions between the aminomethyl lipoate arm and protein H finally disappear, and the aminomethyl lipoate arm departs from protein H, instead of making transient interactions with sites on the surface of protein T.

### Energy landscape and conformational changes during the release process

It is of interest to investigate the energy landscape of the release process and to see if the five conformational states described above can be defined energetically by considering the energy barriers of the process. For this purpose, we calculated changes in the potential of mean force (PMF) for the protein H–T interaction system. The value of PMF is the free energy of a system if it is constrained along with a certain reaction coordinate to a specific value^25^. If the conformations of a protein system can be distinguished using an appropriate coordinate, variations in the PMF will represent the free energy landscape of the system and can provide a basis for calculating free energy differences between conformational states and the barriers between them. PMF can be estimated by calculating the equilibrium probability along the coordinate using techniques such as umbrella sampling simulations^26^.

**Fig. 4:**
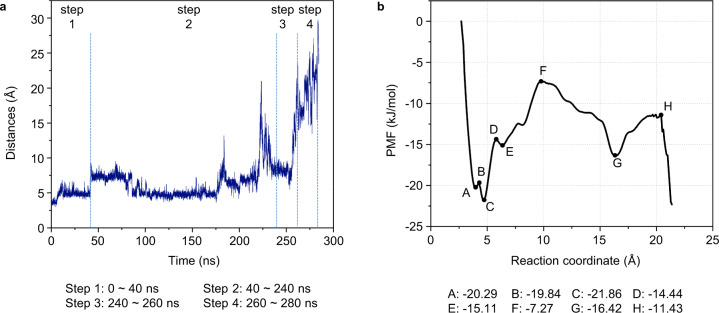
Reaction coordinate and energy landscape of the release process. **a** The distance between the N17 atom of the aminomethyl lipoate arm and the center of mass (COM) of Glu-12 and Glu-14 in protein H as the reaction coordinate and its change a function of time of the induction process of protein T. **b** Potential of Mean Force (PMF) profile of the induction process as a function of the reaction coordinate generated using umbrella sampling simulations and the Weighted Histogram Analysis Method (WHAM).

We used the distance between the atom N17 in the aminomethyl lipoate arm and the COM of Glu-12 and Glu-14 of protein H as a reaction coordinate because of their importance in defining the initial (protected) state of the aminomethyl lipoate arm in the cavity of protein H. The time dependence of this distance during an induction simulation is depicted in Fig. 4a. Consistent with the state transition scheme shown in Fig. 3, four-step changes in the reaction coordinate were observed, the most significant occurring after about 240 ns. The PMF profile of the induction process as a function of the distance (reaction coordinate) generated using umbrella sampling simulations and the Weighted Histogram Analysis Method (WHAM)^27,28^ is depicted in Fig. 4b. Remarkably, the PMF profile also shows four major upward steps, namely A to B, C to D, E to F, and G to H, as implied by our MD simulation of the conformational changes induced in protein H (Fig. 3). Overall, the main energy barrier for the release of the aminomethyl lipoate arm (between the peak value at point F and the valley at point C) is calculated to be 14.6 kJ/mol, which is overcome owing to the induction of protein T. The individual steps are described in more detail below.

**Fig. 5:**
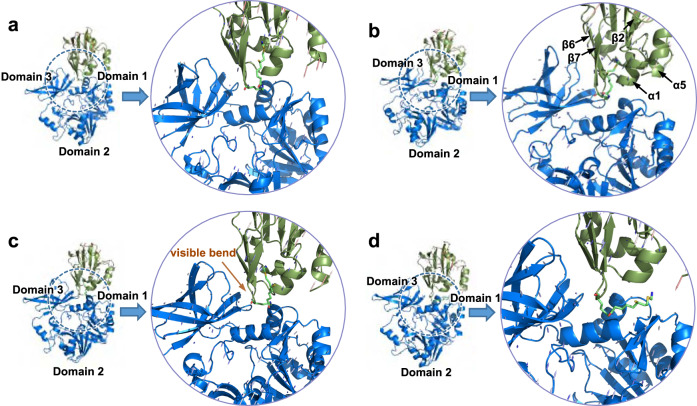
Conformational changes of ecH_int_-ecT interaction in the induction process of protein H by protein T. **a**–**d** correspond to the steps A to B, C to D, E to F, and G to H of Fig. 4, respectively.

Energetically, the system first reaches a pseudostable state at point A. At this point the aminomethyl lipoate arm is locked into a highly stable conformation of protein H. After the binding of protein T induces an interaction between the aminomethyl lipoate arm and protein H, the energy of the system starts to increase until it reaches point B. We collected more than ten frames in the A to B step and drew the conformational changes using Bio 3D^29^. The results are displayed in Fig. 5a. Domain 1 and domain 3 of protein T move in opposite directions, thereby pulling the β7 strand and the α1 helix of protein H in opposite directions. Consequently, the cavity that locks the aminomethyl lipoate arm starts to open. Details of the protein interactions are depicted in Supplementary Fig. 3a.

In the C to D step, the system crosses a second, larger energy barrier. This corresponds to the transition between State II and State III in Fig. 3, during which the aminomethyl lipoate arm starts to leave the cavity. Similarly, we collected more than 40 frames to show the conformational changes in this step (Fig. 5b). Domain 1 and domain 3 of protein T move toward each other. While the β2, β6, and β7 strands, and the α1 and α5 helices of protein H move upward. As a result, residue Ser-67 of protein H forms a hydrogen bond with the aminomethyl moiety which hampers the movement of the aminomethyl lipoate arm. Further induction of protein T makes the aminomethyl moiety loosen itself from its hydrogen bond to Ser-67. The interface interactions of proteins T and H are shown in Supplementary Fig. 3b. In the following E to F step, which corresponds to Step 3 in Fig. 3, the aminomethyl moiety interacts with residue Asp-68 in the cavity of protein H, which becomes loose by the induction of protein T. We collected more than 60 frames to show conformational changes at the interface between proteins H and T (Fig. 5c). In this step, the conformational changes at the protein interface are similar but not as dramatic as those that occur in the C to D step. In the E to F step, protein H shows a visible bend in the β7 strand, a significant difference that distinguishes ecH_red_ from ecH_int_. Okamura-Ikeda^30^ reported this bend previously and conjectured that it triggers a crucial change when ecH_int_ binds to protein T. Thus, the bend in strand β7 is an important sign that marks the release of the aminomethyl lipoate arm from the cavity. The corresponding protein interface interactions are shown in Supplementary Fig. 3c. The last energy barrier to be overcome is the hydrogen-bond interaction between the aminomethyl moiety and residue Tyr-70 of protein H in step 4. At point H, the aminomethyl lipoate arm has completely escaped from the cavity of protein H. We collected more than 40 frames to show the conformational changes on the protein interface (Fig. 5d). In this step, domains 1 and 3 of protein T move in the opposite direction similarly to the motion observed in the A to B step, and protein H moves toward protein T, so that the aminomethyl lipoate arm completely leaves the cavity and binds to protein T. The relevant protein interface interactions are shown in Supplementary Fig. 3d.

### Experimental tuning of the release process

**Fig. 6:**
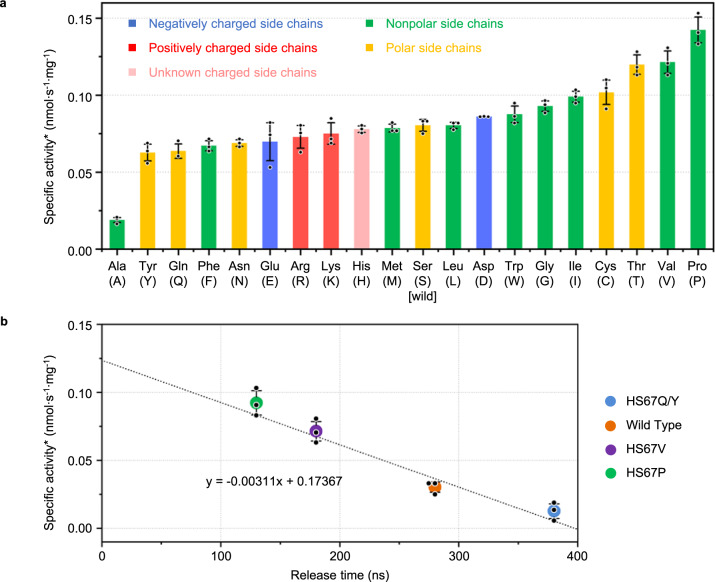
Effects of mutations of H protein on glycine cleavage system (GCS) activity and its correlation with simulation results. **a** The activity of glycine cleavage catalyzed by the wild type (HSer-67) and its mutants of protein H. **b** Correlation between enzyme activity and the calculated time for the release of the aminomethyl lipoate arm from the cavity in the wild type and mutants of protein H.

Based on our analysis of the simulations, it is clear that the second step (Step 2), i.e., from State II to State III in Fig. 3 and point C to point D in Fig. 4, represents the limiting step that takes the longest time and possesses the highest energy barrier during the release of the aminomethyl lipoate arm. Residue Ser-67 of protein H is thus a key player in the release process. In order to tune the release process, saturation mutagenesis of Ser-67 was carried out. The effects of the mutations were first evaluated by measuring their glycine cleavage activities (*n* = 3 independent experiments). The results are shown in Fig. 6a, in comparison to that for wild-type protein H (HSer-67). GCS activity was altered both positively (up to 80% activity increase) and negatively (up to 20% activity decrease) by the mutations, indicating that residue Ser-67 of protein H plays an essential role by controlling the release of the aminomethyl lipoate arm from the cavity of protein H.

### Relationship between release time and enzyme activity

**Fig. 7:**
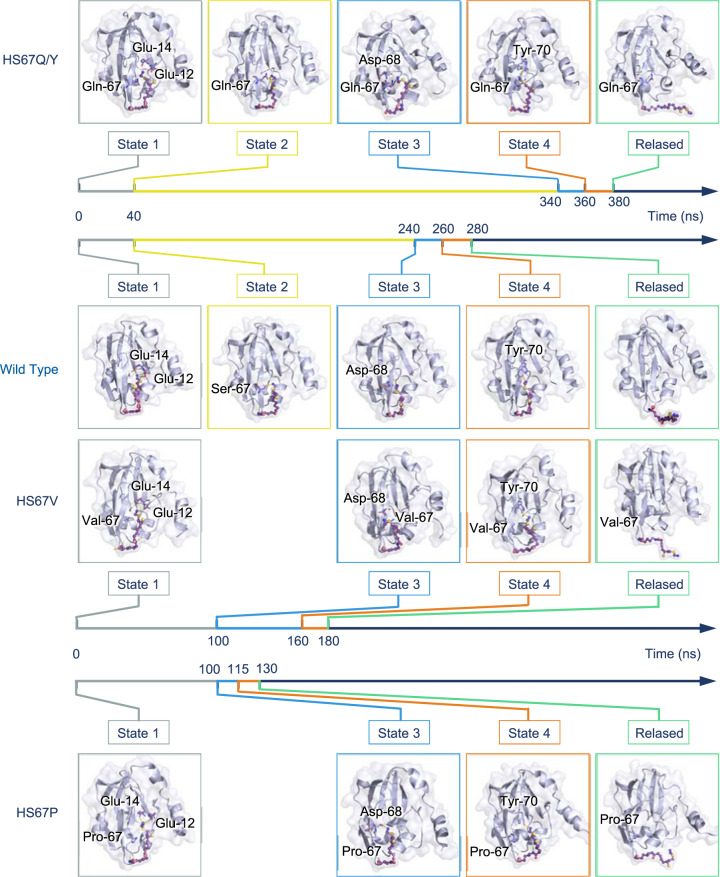
Different states and the time spans in the different steps of the mutants in comparison with those of the wild-type protein H. The time spans (in ns) in the different states of the mutants HS67Q/Y and HS67V are given below the corresponding panels with the MD graphs. Those for the wild type and the mutant HH67P are given above the corresponding panels.

In order to obtain more quantitative and mechanistic information about the activity change that resulted from the mutations, we carried out MD simulations for the following four mutants: HS67Q, HS67Y, HS67V, and HS67P (the former two with clearly decreased activity and the latter two with increased activity, as shown in Fig. 6a). The conformations of the four mutants were balanced for 60 ns at first for stability and then simulated for interactions with protein T for 500 ns. The MD trajectories were analyzed in a similar way to that described for the wild type above. Snapshots of the simulation results are depicted in Fig. 7. In the simulations of the HS67Q-T complex, the aminomethyl lipoate arm passes through four states before it is released, which is consistent with the trajectory described for the wild type (Fig. 3). However, the aminomethyl lipoate arm stays in Step 2 for about 300 ns in the mutant (vs. 200 ns in the wild type). The other steps are not affected. The total release time is thus increased from 280 ns to 380 ns, leading to a decreased enzyme activity. Similar results are obtained for the mutant HS67Y. Here, the benzene ring of Tyr-67 could form a π-cation interaction with the aminomethyl moiety, which could be responsible for keeping the aminomethyl lipoate arm in Step 2 for 300 ns. On the other hand, in our simulations of HS67V-T and HS67P-T, the aminomethyl lipoate arm was found to move directly from State I into State III without going through State II, meaning that the time-consuming Step 2 experienced by the wild type can be skipped over. In these cases, the total time required for moving from State I to State III lasts for only 100 ns instead of 240 ns for the wild type. Although for HS67V Step 3 lasts somewhat longer than for the wild type, the overall time (200 ns) is still much shorter, leading to increased enzyme activity. The most significant effect is observed for the mutant HS67P. Here, Step 3 and Step 4 are also shortened, leading to an overall release time of only 150 ns. It is interesting to note that the HS67P mutation leads to a large conformational change in strand β7 of protein H. This seems to bend earlier than in the wild type and other mutants, leading to a rapid release of the aminomethyl lipoate arm and to the highest overall enzyme activity among all the mutants tested so far.

## Discussion

GCS is a fascinating multi-enzyme complex that possesses a shuttle protein, which contains a lipoate swinging arm. Protection of the aminomethylated lipoate arm in the hydrophobic cavity of protein H and its release induced by protein T are key processes of this molecular machine that to date have been poorly understood. Here, we first present a detailed molecular dynamics (MD) analysis of the interactions within the *E. coli* H–T protein complex that governs the induced release of the aminomethyl lipoate arm from a stable state in the cavity of protein H. Four key steps in the release process are revealed and their molecular dynamics are studied in detail, highlighting Ser-67 in the cavity of protein H as making a major contribution to the energy barrier that protects the lipoate-attached reaction intermediate. Saturation mutagenesis of this key residue led to the identification of mutants of protein H with significantly accelerated or delayed release of the aminomethyl lipoate arm (Fig. 6). This fine-tuning of a key player in C1 metabolism could have significant applications in both biotechnology and biomedicine^31^.

MD simulations were used to examine the molecular basis for differences in the enzyme activity of four selected mutants (Fig. 7). Drastic changes were found to be associated with the modulation of Step 2 (duration 300 ns in HS67Q/Y vs. 200 ns in the wild type; it is completely skipped over in HS67V/P), whereas the other steps were only slightly or moderately affected. It is of interest to have a more mechanistic understanding of these mutants based on the properties of the side chains that were altered. The results can be roughly interpreted in terms of the physiochemical properties (side-chain charge/polarity and length) of the mutated amino acids and their potential effects on interactions with the nitrogen atom (N17, positively charged) in the aminomethyl lipoate arm, though there are exceptions. Substituting Ser-67 with negatively charged residues such as glutamic acid could result in the enhanced attraction of the positively charged N17 which conceivably could hamper the release of the aminomethyl lipoate arm and lead to reduced enzyme activity. Interestingly, mutations in which Ser-67 is changed to a positively charged residue, such as arginine and lysine, also had a negative effect on the enzyme activity. We speculate that the electrostatic repulsion between N17 and positively charged residues could make the aminomethyl lipoate to stay longer in Step 1, and difficult to reach State II. This can hamper the release of the aminomethyl moiety by hindering the crossing of the energy barrier. In addition, enzyme activity appeared to decrease as the length of the side chain of the amino acid increased. For instance, the side chain of glutamic acid (E) is longer than that of aspartic acid (D) and the enzyme activity of HS76E was lower than that of HS67D. As shown in Fig. 3(II), Ser-67 in the wild-type is also a polar residue, and the hydrogen bond it forms with N17 slows the release of the aminomethyl lipoate arm. However, mutations in which the serine is replaced by a more polar residue, such as glutamine (Q) and asparagine (N), could form stronger hydrogen bonds with N17 and thereby slow the release process, leading to lower enzyme activity. In general, most of the mutants with nonpolar residues showed higher enzyme activity than the wild-type, especially HS67P and HS67V. Thus, abolishing polar interactions with N17 could accelerate the release process. Mutations of Ser-67 to tyrosine (Y) and phenylalanine (F) led to significantly lowered enzyme activity. The benzene ring in these side chains could create steric hindrance and/or form a π–cation interaction with the aminomethyl moiety. Both actions would slow down the release of the aminomethyl lipoate arm.

It should be pointed out that the interpretation of the mutation results in terms of the physiochemical properties of the residues should be considered with caution and there are exceptions. For example, the effect of S67T seems to be contradictory. As serine to threonine mutation only changes the size of the side chain, it can be considered to be a more direct indication of the steric effect on the activity. However, S67T promotes the activity. Also, glycine, alanine, and valine are usually considered as one group when categorizing the amino acids. They are the very typical nonpolar residues, and the steric hindrance of the side chain increases gradually. However, they have different even opposite effects on the activity. We would like to emphasize that the enzyme activity measured in this work represents the overall activity of the GCS, not only the aminomethyl transfer step since no direct and efficient method is available to quantify the rate of this single step. We suspect that the mutation of S67 may also affect the self-protection process of the aminomethyl lipoate (the opposite of the release process). Furthermore, the mutations could affect the overall structure and dynamics of protein H. For a more complete picture and consistent interpretation, the self-protection process and the overall protein dynamics should be also studied.

Irrespective of the detailed mechanism(s), it is interesting to note that there was a strong correlation between the experimentally measured glycine cleavage activity of GCS and the time calculated for release of the aminomethyl lipoate arm from the cavity of protein H (Fig. 6b). This underlines on one hand the usefulness of the 3D model we established for H_int_ and the subsequent MD simulation methods used to study its interactions with protein T. On the other hand, our results strongly suggest that under the experimental conditions we have tested, the release of the aminomethyl lipoate arm constitutes the limiting step that controls the overall activity of GCS. According to our best knowledge, this is for the first time that such a strict correlation between enzyme activity and a parameter derived from MD simulations of the interaction between two proteins has been shown for a molecular shuttle.

It is noted that only a single long time simulation was performed for the wild type ecH_int_ in this work due to limitations of computational resources under the present special circumstance. Although a single long time simulation could provide insights into global rearrangements^32^, different paths caused by the ruggedness of the energy landscape could be better explored with replicas^33^. A single trajectory is likely to be trapped in a local minimum. If the number of replicas is enough, other replicas could continue to explore the remaining solution space. The consideration of differences in average descriptors of multiple replicas can achieve more reliable results^34^. Nevertheless, we have run ten 1 ns simulations with different temperatures and starting seed to get a representative structure for the long simulation. We believe that our key conclusion (the division of the process into four steps and key residue(s) for each of them) will not be significantly affected by the limited long time simulations. Importantly, we have backed up our conclusion by analyzing the energy landscape and conformational changes during the release process and by mutations of a selected key residue.

The quantitative approach presented in this study can be used to guide further targeted engineering of GCS to modulate its activity and perhaps even reverse its direction to facilitate biosynthesis. A similar approach may also be used to study and engineer molecular shuttles involved in other essential biological processes, such as pyruvate and 2-oxoglutarate metabolism or fatty acid synthesis. Rational manipulation of these enzyme systems could have a tremendous impact on the development of more efficient biomanufacturing processes using sustainable resources including CO_2_. Further applications could include the development of novel drugs and therapies for treating challenging diseases, such as obesity^35^, neurodegeneration^36^, or cancer^37^. The predictable and controllable tuning of C1 energy metabolism reported here represents a significant step toward this end.

## Methods

### ecHint model building

**Fig. 8:**
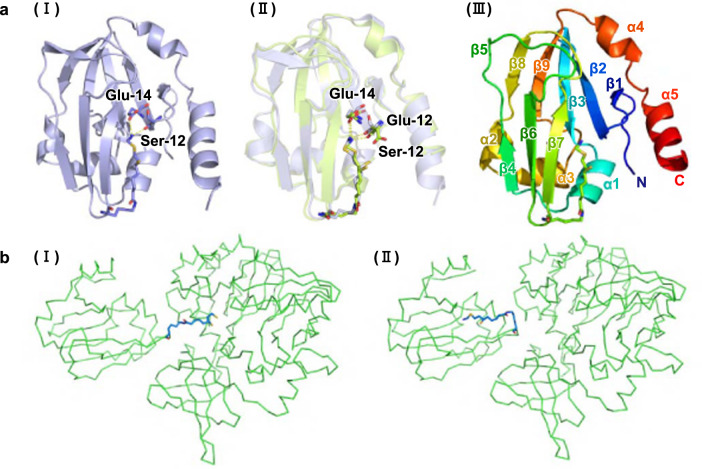
Building of 3D structure ecH_int_ and ecHint-ecT complex models. **a** The building of ecH_int_ model based on the 3D structures of peaH_int_ and ecH_apo_. (I) 3D structure of peaH_int_; (II) alignment between the 3D structures of peaH_int_ and ecH_apo_; (III) built ecH_int_ model for molecular dynamic simulations. **b** (I) The ecH_red_-ecT complex model from Okamura-Ikeda et al.^30^, in which dihydrolipoic acid is inserted into protein T, representing the state where the protein H–T interaction induced release process of aminomethyl lipoate is completed and the aminomethyl moiety has reacted with THF. (II) The ecH_int_–ecT complex built in this work, in which the aminomethyl lipoate arm is protected in the cavity of protein H, representing the state where the induction process has not yet started.

In this work, we chose to study protein H from *E. coli* (ecH) for its importance as a chassis for synthetic biology^31^. We have used the numbering of amino acid residues of the *E. coli* H-protein according to its crystal structure and usage in prior literature^30^. However, in NCBI and other protein databases there is an additional amino acid methionine (M) at the beginning of the H-protein sequence. This residue is not present in the reported crystal structure. However, no direct experimental data are available for the structure of the intermediate form of ecH (ecH_int_). Fortunately, structures for the apo-form of protein H from *E. coli* (ecH_apo_) and the intermediate form of protein H from *Pisum sativum* (peaH_int_) had been solved previously (PDB IDs 3A7L^38^ and 1HTP^2^). Structural alignment revealed that the root-mean-square deviation (RMSD) of the tertiary structures of peaH_int_ and ecH_apo_ was very low (1.17 Å), suggesting that the tertiary structures of the two proteins are very similar to each other. For residues that line the hydrophobic cavity^2^ that traps the aminomethyl lipoate arm, the RMSD drops as low as 0.69 Å (Supplementary Fig. 5). The amino acid sequence alignment of cavity regions of the two proteins showed an identity of 60%. An alignment of protein H sequences from various species is provided in Supplementary Fig. 4. Hence, the spatial position of the lipoate arm could be transferred from peaH_int_ to ecH_int_. Based on the tertiary structures of ecH_apo_ and the spatial position of the lipoate arm in the cavity of peaH_int_, we built a tertiary structure model of ecH_int_, as shown in Fig. 8a. This ecH_int_ model was then used in the molecular dynamics simulations throughout this work.

### The building of the ecHint-ecT complex model

Despite several available experimental studies of the interaction between ecH_int_ and ecT^22^, no structural data was available for the ecHint and ecT complex^23^. On the other hand, Okamura-Ikeda et al.^30^ have published a structure of the ecH_red-_ecT complex (Fig. 8b(I)), which provided us with information about the protein interaction interface between protein H and protein T. By referring to this information we built a model of the ecH_int_–ecT complex (Fig. 8b(II)) for the molecular dynamics simulations carried out in this work.

### Simulation setup

The molecular dynamics simulations and analysis were performed using the GROMACS-2016 package^39^ with the Amber14 force field^40^. The parameters including charges (AM1-bcc) and atom types for the lipoate arm were generated using Antechamber provided in Ambertools17^41^. The visual molecular dynamics^42^ (VMD) program and PyMOL^43^ were used for visualizations. For each simulation, the system with 1.0 nm of padding was filled with TIP3P water molecules and neutralized with Na^+^ counter ions. Each system was minimized using the steepest descent of 5000 steps and conjugate gradient methods of 5000 steps to eliminate any overlap or clash between the atoms. Convergence was reached when the maximum force was not greater than 1000 kJ mol^−1^ nm^−1^ at any atom. Then the system was heated up to 300 K using the Velocity-rescale method^44^. At this stage, position restraints on the heavy atoms of the protein allowed the surrounding water molecules to relax. After the system temperature was stabilized, the system was equilibrated at a constant pressure of 1 bar using the Parrinello-Rahman method^45^. A simulation was run for 2 ns until the density was stable. After that, simulations were performed using an integration time step set to 2.0 fs and the Particle Mesh Ewald (PME) method for long-range electrostatics and a 1.0 nm cutoff for short-range electrostatics. All hydrogen-bond vibrations were constrained using the linear constraint solver (LINCS) algorithm.

For each system, including the wild type and mutants, tentrajectories for 1 ns starting from varied target temperatures and random number seeds were simulated. Afterward, clustering analysis^46^ was used to choose a “representative” structure. The “representative” structure is the conformation with the smallest average root-mean-square deviation from the centroid of the largest population. Subsequently, we simulated the “representative” structure for the following simulations to obtain the trajectory.

### Umbrella sampling

To generate the PMF profile of the induction process as a function of the distance (reaction coordinate) (Fig. 4b), umbrella sampling simulations were carried out based on Fig. 4a. For this purpose, 34 evenly spaced reference points between 3.75 Å and 21.12 Å were chosen as initial conformations. Each conformation was simulated for 200 ps after equilibration at constant pressure, followed by a 20 ns umbrella sampling simulation with a time step of 2 fs.

### Mutagenesis experiments and analysis

Primers for the cloning and expression of genes for the mutants are listed in Supplementary Table 2. The plasmid construction for gene expression was accomplished using standard techniques^47^. PCR-based site-directed mutagenesis was accomplished according to the protocol provided in a Mut Express II Fast Mutagenesis kit V2 (Vazyme, Jiangsu, China). Recombinant cells were cultured at 37 °C in 100 mL of Luria–Bertani (LB) medium containing 50 μg/mL kanamycin. Expression was induced using 0.1 mM isopropyl β-D-1-thiogalactopyranoside (IPTG) added at about OD_600_ = 0.7. The wild type and mutants of protein H were expressed with 150 µM lipoic acid added exogenously to obtain the ecH_ox_ form. After induction, the cells were cultured at 30 °C for 12 h and harvested by centrifugation at 4000 × *g* for 30 min at 4 °C. Pellets were resuspended in a Tris-HCl buffer (50 mM, pH = 7.5) and the cells were broken using a Xinzhi JY92-IIN Ultrasonic Homogenizer in an ice bath. Then the supernatant was collected by centrifugation at 4000 × *g* for 30 min at 4 °C.

All the proteins were purified using His-tag affinity chromatography (ÄKTA, GE Healthcare, USA) equipped with a nickel column (HisTrap^TM^ HP, 5 ml). Buffer A containing 500 mM NaCl, 50 mM Tris-HCl and 20 mM imidazole (pH = 7.4) was used to elute non-target proteins, and buffer B containing 500 mM NaCl, 50 mM Tris-HCl, and 500 mM imidazole (pH = 7.4) was used to elute the target protein adsorbed in the nickel column.

The activity of the glycine cleavage enzyme was determined by measuring the reduction of NAD^+^ at 340 nm. The reaction mixture (200 μL) contained 1 mM NAD^+^, 1 mM THF, 0.1 mM PLP, 6 µM protein P, 13 µM protein T, 8 µM protein L, 100 mM Tris-HCl (pH 7.0), and 6 µM of either the wild-type protein H or one of its mutants. The components were premixed and centrifuged before the reaction was initiated by adding 1 mM glycine. One enzyme activity unit (U) was defined as the amount of protein H that produces 1 μmol of NADH per min.

### Statistics and reproducibility

3D structures of the protein used at atomic resolution were deposits in the Protein Data Bank. RMSD values of the structures in the trajectory were calculated using the gmx_rms command line and by taking the first frame as the reference. The distances of the reaction coordinate were calculated using the gmx_distance command line. The hydrogen bonds were counted using the gmx_hbond command line. PMF as a function of the reaction coordinate was obtained using the WHAM. NNK degenerate codon was used to construct a mutant library. Enzyme activities were measured by three independent experiments and averaged for the report. Individual data points are added in the graphs, and error bars are defined by the standard deviation.

## Supplementary information

Supplementary Information

Description of Additional Supplementary Files

Supplementary Data 1

## Data Availability

Major data generated and analyzed during this study are included in the article and its Supplementary Information. The source data underlying the graphs and charts presented in the main figures are available as Supplementary Data. Other datasets generated and analyzed during the study are available from the corresponding author on reasonable request.
