# Supplementary material for: Structure-based dynamic analysis of the glycine cleavage system suggests key residues for control of a key reaction step
Source: Commun Biol. 2020 Dec 11;3:756. doi: 10.1038/s42003-020-01401-6 (PMC7733448; doi:10.1038/s42003-020-01401-6)
Supplement: Supplementary file 1 — Supplementary Information [file 42003_2020_1401_MOESM1_ESM.pdf]

Supplementary Materials

Structure-based dynamic analyses of the glycine cleavage system suggests key residues for control of a key reaction step

Han Zhang[1], Yuchen Li[1], Jinglei Nie[1] Jie Ren[1,3*], and An-Ping Zeng[1,2*]

[1]Beijing Advanced Innovation Center for Soft Matter Science and Engineering, Beijing University of Chemical Technology, North Third Ring Road 15, Chaoyang District, 100029, Beijing, China

[2]Institute of Bioprocess and Biosystems Engineering, Hamburg University of Technology Denickestrasse 15, D-21073 Hamburg, Germany

[3]State Key Laboratory for Biology of Plant Diseases and Insect Pests/Key Laboratory of Control of Biological Hazard Factors (Plant Origin) for Agri-product Quality and Safety, Ministry of Agriculture, Institute of Plant Protection, Chinese Academy of Agricultural Sciences, Beijing 100081, China

[*] Corresponding author

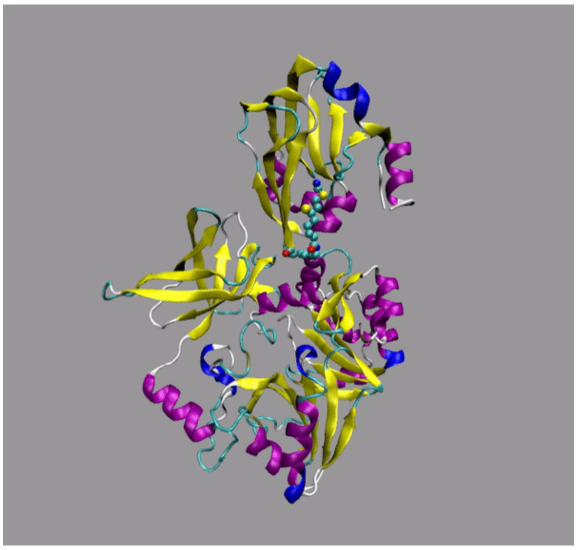

Supplementary Figure 1. Dynamics of release of the aminomethyl lipoate arm from the cavity of the protein H induced by protein T (3D animation).

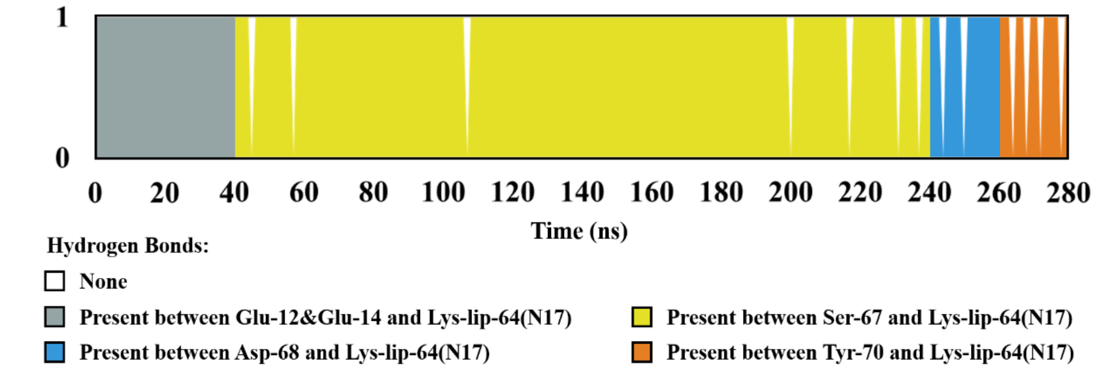

Supplementary Figure 2. Formation of charged hydrogen bonds over the simulation time.

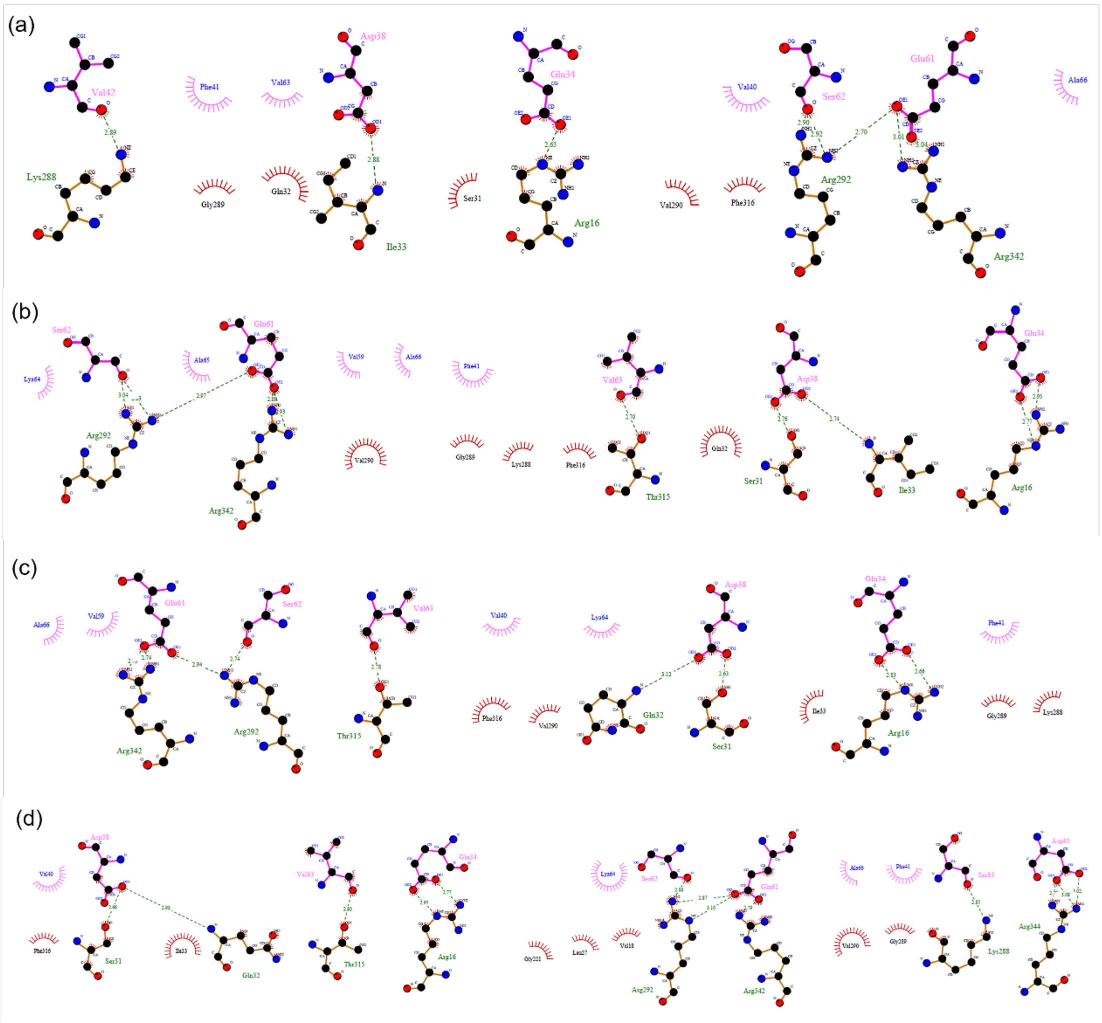

Supplementary Figure 3. Detailed interactions of structural interface between protein H and the protein T. **(a)** the step AB **(b)** the step CD **(c)** the step EF **(d)** the step GH

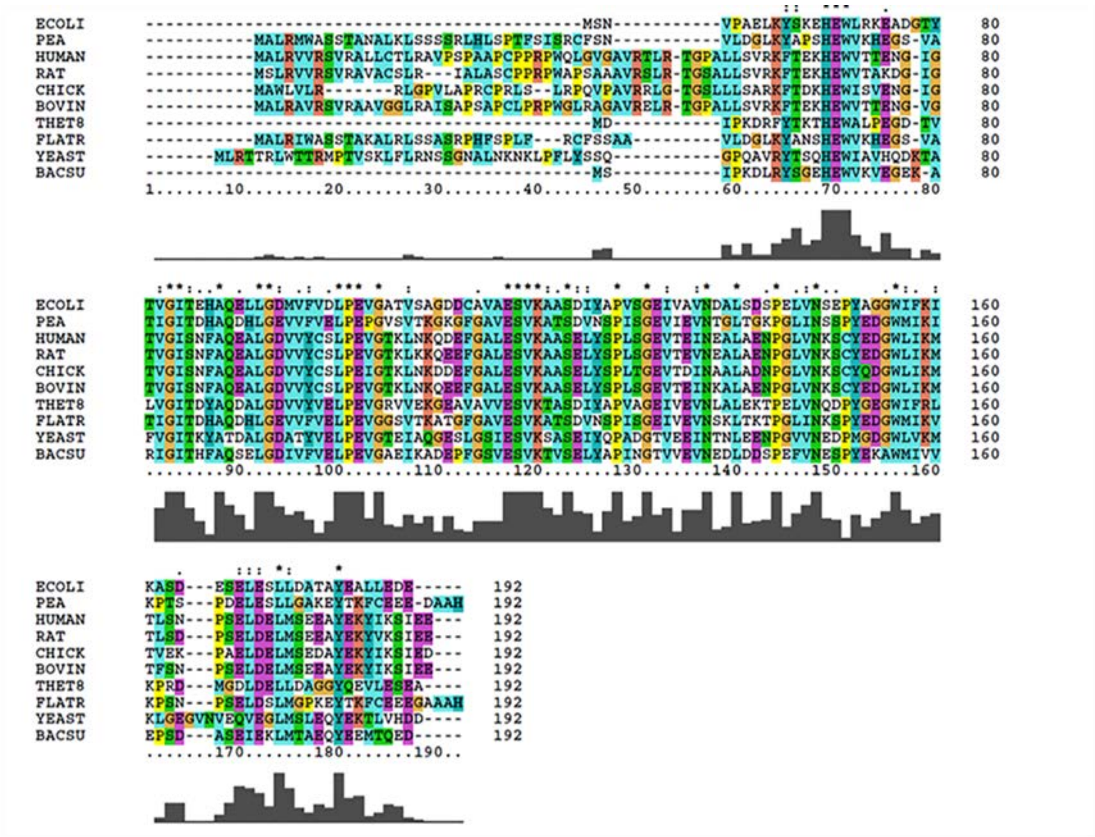

Supplementary Figure 4. The sequence alignments of protein H from various species by ClustalX21

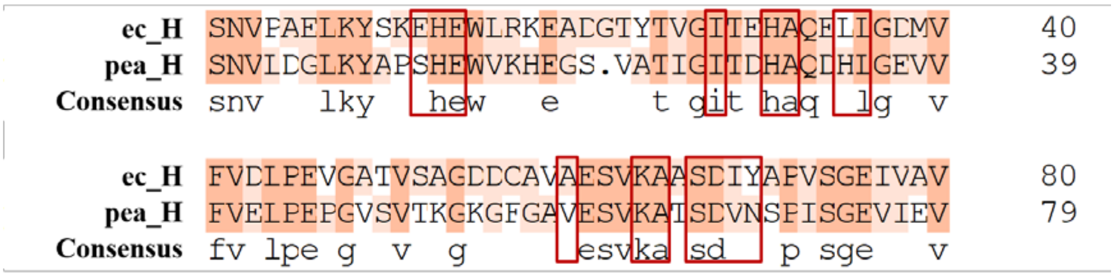

Supplementary Figure 5. The residues (in red frame) composing the hydrophobic cavity in ecH_{apo} and $peaH_{int}$.

Supplementary Table 1. Charged hydrogen bonds formed in the release process of the aminomethyl lipoate arm (referred as Lys-64-lip) (see Figure 3 for details).

"Frames" stands for the number of frames in which the formation of a hydrogen bond between the acceptor of a residue in the cavity and the donor prevails; and "Time" gives the duration between the formation and breakage of a hydrogen bond.

Acceptor	Donor	Frames	Time (ns)
Leu-35	Lys-64-lip(N6)	14002	280.04
Ser-67	Lys-64-lip(N17)	10761	215.22
Asp-68	Lys-64-lip(N17)	1967	39.34

Tyr-70	Lys-64-lip(N17)	1044	20.88

Supplementary Table 2. Primers for the cloning and expression of gene for the mutants

Primer	Oligonucleotide Sequence
HS67-saturated mutation-fwd	5’- CGAATCAGTAAAAGCGGCGNNKGACATTTATG -3’
HS67-saturated mutation -rev	3’- GCCAACGGCTTAGTCATTTTCGC -5’
