# Supplementary material for: Structure-based dynamic analysis of the glycine cleavage system suggests key residues for control of a key reaction step
Source: Commun Biol. 2020 Dec 11;3:756. doi: 10.1038/s42003-020-01401-6 (PMC7733448; doi:10.1038/s42003-020-01401-6)
Supplement: Supplementary file 2 — Description of Additional Supplementary Files [file 42003_2020_1401_MOESM2_ESM.pdf]

**Description of Additional Supplementary Files**

**File name:** Supplementary Data 1

**Description:** Source data underlying the graphs in the following main figures: Fig. 2a, Fig. 2c, Fig. 4a, Fig. 4b, Fig. 6a, Fig. 6b.
